# Colonic Lipoma Inducing Bowel Intussusception and Transanal Prolapse: A Presentation of a Rare Case

**DOI:** 10.7759/cureus.71291

**Published:** 2024-10-12

**Authors:** Beatriz Vila Ariza, Fernando Carvajal Lopez, David Negre Parra, Genesis Daniela Parra Eslava, Natalia Uribe Quintana

**Affiliations:** 1 Digestive Surgery, Centro de Estudios Universitarios (CEU) Cardenal Herrera University, Valencia, ESP; 2 Digestive Surgery, Hospital Arnau de Vilanova, Valencia, ESP

**Keywords:** benign colonic tumors, colonic intussusception, colonic lipoma, colonic obstruction, transanal prolapse

## Abstract

Colonic lipomas are rare benign tumors that may appear throughout the entirety of the gastrointestinal tract, with a predisposition to appear in the colon. Patients with colonic lipoma are typically asymptomatic, making their diagnosis rare and incidental. This case report intends to investigate and clarify the decision-making process regarding surgical segmental colonic resection versus local excision via the study of a 48-year-old man with ulcerative colitis diagnosed in 2006, undergoing treatment with infliximab and without exacerbations since 2010. The patient presents with anal prolapse and colonic obstruction due to intussusception. After performing a CT scan with contrast, an intramural lipoma in the sigmoid colon measuring 5.5×4.7×4.6 cm was revealed. An elective oncologic sigmoid resection was the treatment of choice with regard to its size, location, and associated symptoms, especially when presenting complications like intussusception. Such a decision allowed for an improvement in pain control, shorter hospitalization, and faster recovery. The decision was also taken considering the safety risks regarding the size of the lesion and the presence of the intussusception.

## Introduction

Lipomas are characterized as slow-growing, non-epithelial benign neoplasms composed of adipose tissue [1,2]. These tumors, slightly more frequent in women [1] (with a 57% incidence rate) between the ages of 40 and 70, may manifest at various anatomical sites, typically situated between the dermal layer and the underlying subcutaneous musculature. Rarely, they may also occur along the entirety of the gastrointestinal tract, with an incidence rate of 0.035-4.4%, most frequently located in the colon [1]. 

Intestinal intussusception, defined as the telescoping of one segment of the bowel into an immediately adjacent segment, is a very uncommon occurrence in adults compared to children, accounting for only 5% of intussusceptions [3]. When associated with lipomas, it can lead to intestinal occlusion and vascular compromise. 

In the following case report, we present a sigmoid intussusception caused by a colonic lipoma presented with transanal protrusion treated with surgical resection, highlighting the decision-making process regarding surgical segmental colonic resection versus local excision.

## Case presentation

The patient was a 48-year-old male in treatment with infliximab for ulcerative colitis, diagnosed in 2006, along with asymptomatic ankylosing spondylitis. The patient's last exacerbation was in 2010, and since then, he has been asymptomatic. The patient consulted in the emergency room for rectal mass prolapse causing colonic obstruction.

The diagnostic steps started with a physical examination that revealed a soft mass of 5 cm in diameter with normal mucosa prolapsed through the anus without signs of tissue ischemia. The following step taken was a series of blood analyses that presented with no irregularities; results are displayed in Table 1.

**Table 1:** Blood test results All are within the normal reference range. INR: international normalized ratio

Blood test	Patient values	Normal values
Hemoglobin	13.5 g/dL	13.5-17.5 g/dL
Leukocytes	6.64×10^9^/L	4-11.5×10^9^/L
Neutrophils	57.3%	40-74%
Platelet count	268×10^9^/L	150-400×10^9^/L
Quick index	95%	70-120%
INR	1.04	0.90-1.15
Creatinine	0.7 mg/dL	0.7-1.3 mg/dL
Procalcitonin	0.03 ng/mL	<0.05 ng/mL
C-reactive protein	1.3 mg/dL	<3 mg/dL

**Figure 1:**
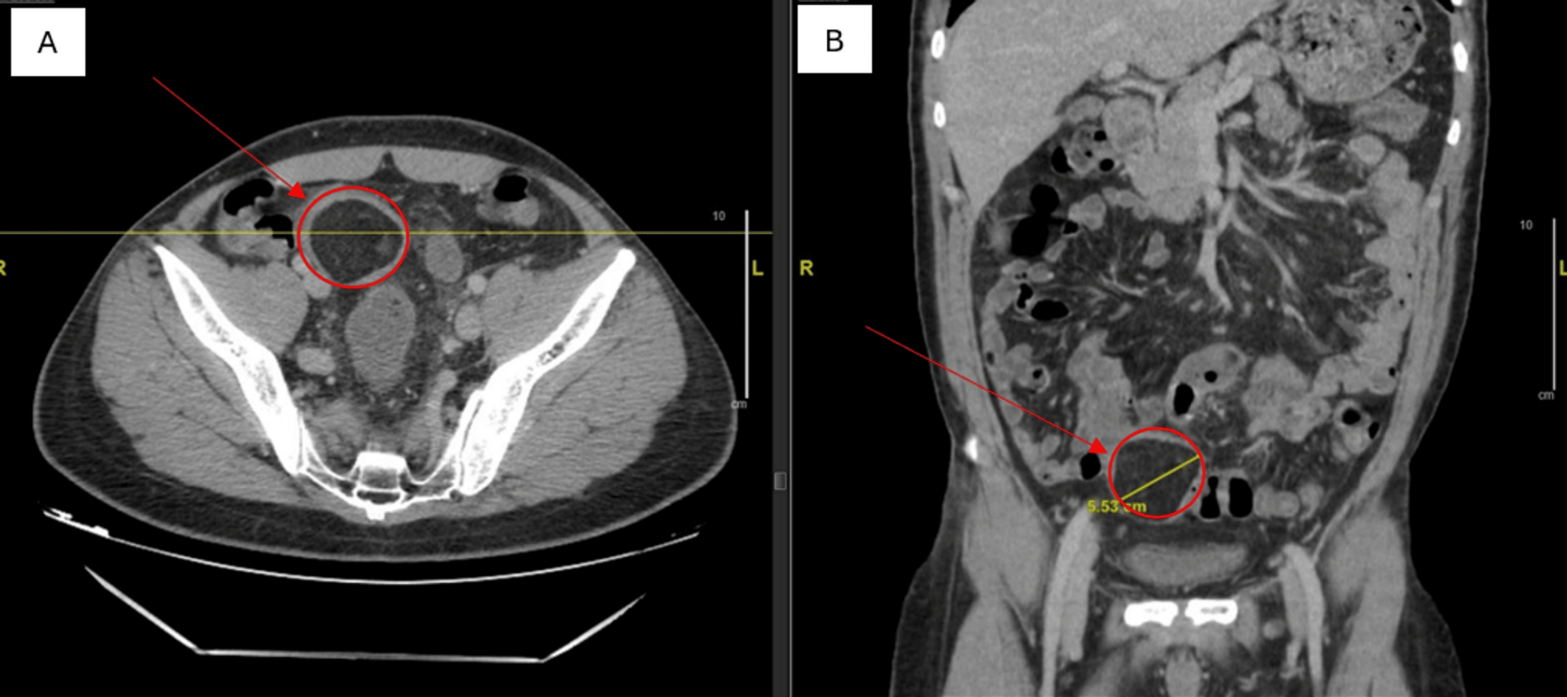
CT scan revealed an intramural lipoma in the sigmoid colon that measured 5.5×4.7×4.6 cm Panel A is an axial slice of the CT scan. Panel B is a coronal slice of the CT scan. In both panels A and B, the lipoma is indicated within the red circle and arrow.

**Figure 2:**
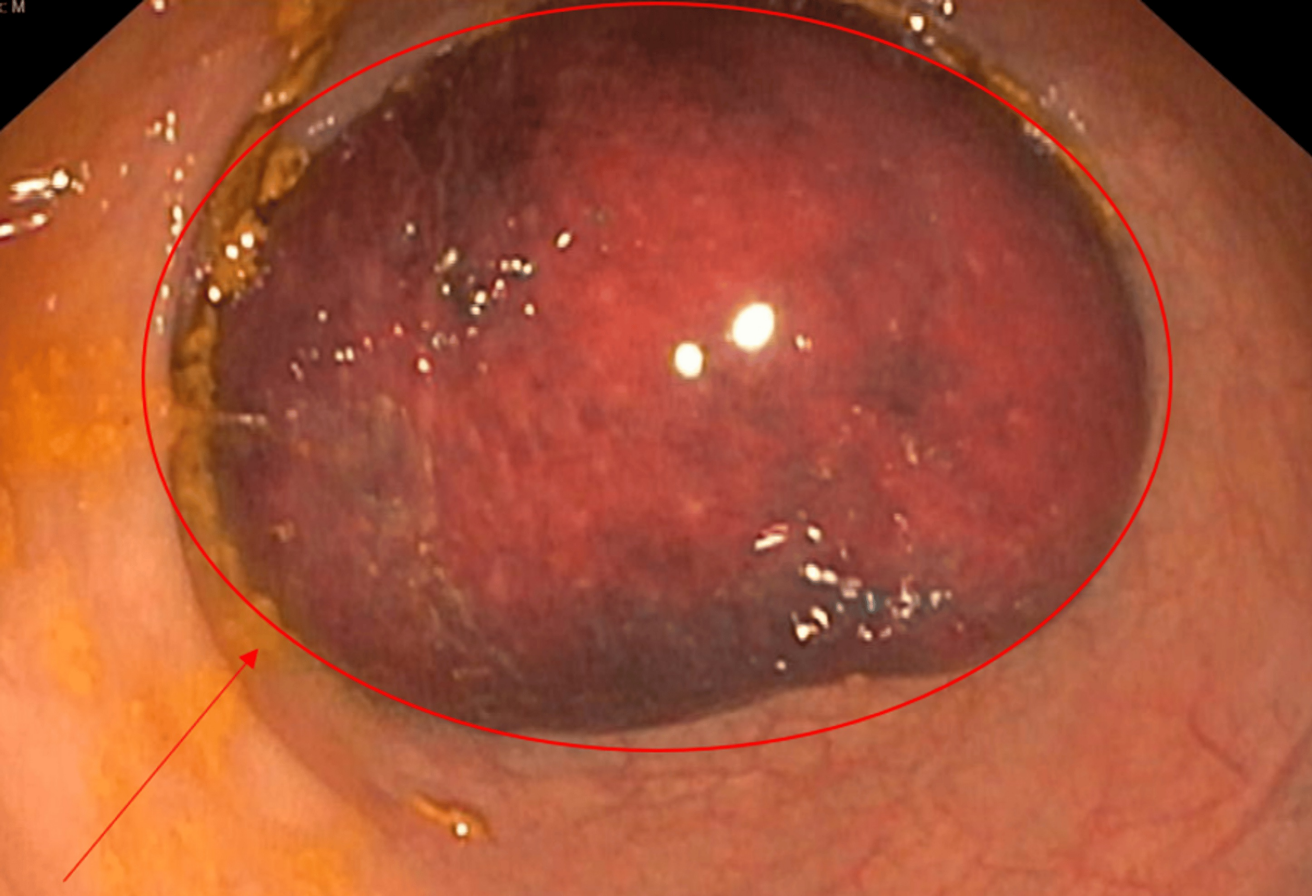
Colonoscopy described a voluminous submucosal tumor with congestive mucosa suggestive of lipoma that caused luminal obstruction The lipoma is indicated within the red circle and arrow.

The patient was admitted to the hospital to further study the case and determine elective treatment. Initially, we performed a digital mass reduction without complications. A CT scan with contrast was then performed and revealed an intramural lipoma in the sigmoid colon that measured 5.5×4.7×4.6 cm (Figure 1), without associated intussusception but with inflammatory changes in the sigmoid fat tissue and colonic wall, suspected to be secondary to prior intussusception. A colonoscopy described a voluminous submucosal tumor with congestive mucosa suggestive of a lipoma that caused a luminal obstruction (Figure 2).

**Figure 3:**
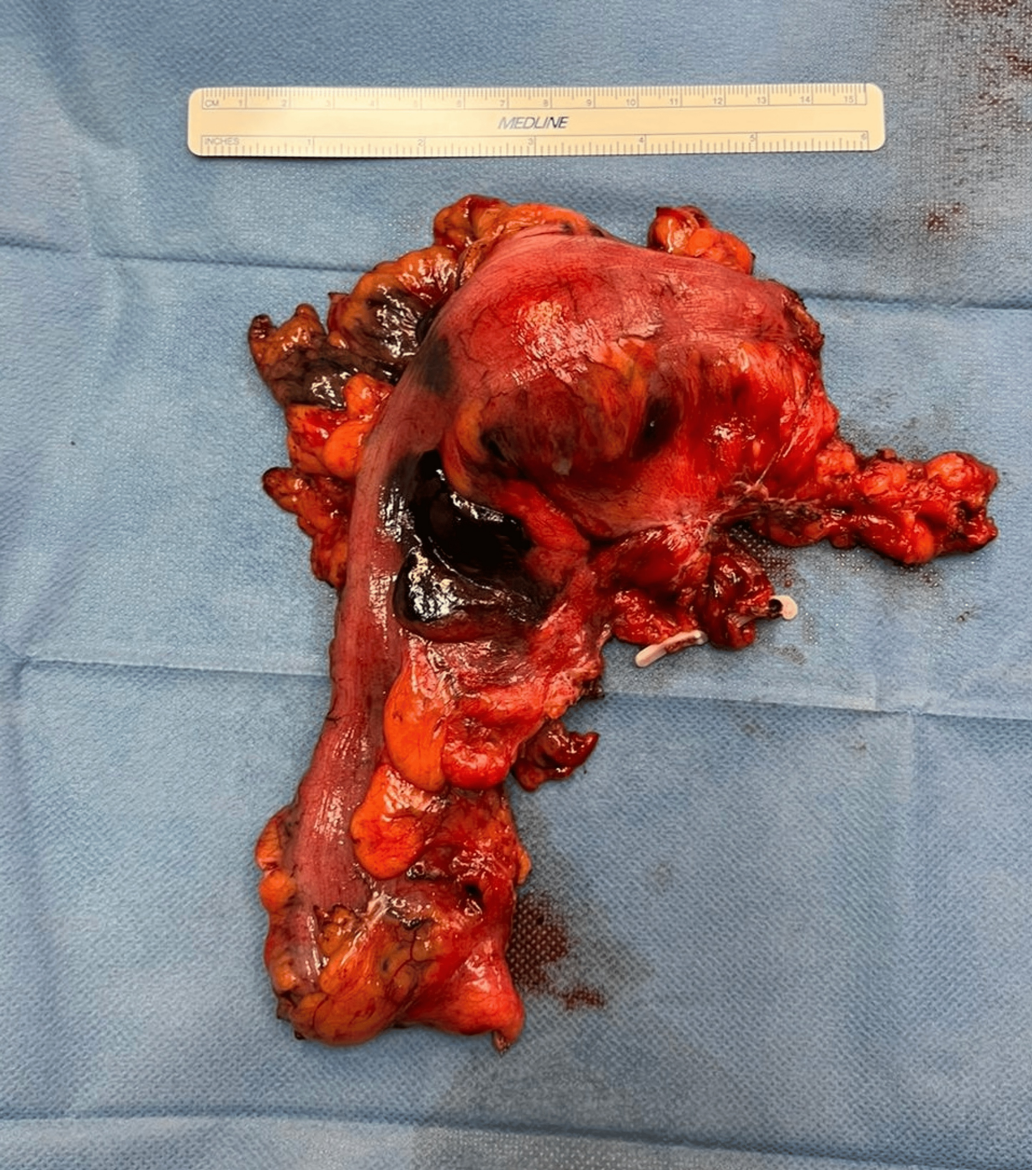
A laparoscopic oncologic sigmoidectomy was performed

Based on the patient's asymptomatic case of ulcerative colitis, good pharmacological management, and lack of disease evolution in routine revisions, a laparoscopic oncologic sigmoidectomy was performed. There were no intraoperative incidences (Figure 3), and the patient was discharged on the fourth day with no postoperative complications. Histopathological exam informed colonic submucosal lipoma with normal lymph nodes. A routine follow-up revision six months later showed no signs of complications.

## Discussion

Colorectal lipomas are a rare benign pathology with a nonspecific array of symptoms that vary depending on the size, location, and morphology of the tumor, with the diagnostic process relying on colonoscopy, imaging techniques, and pathological analysis [1,4].

These benign tumors typically present as soft, mobile masses that rarely associate with symptoms, making diagnosis rare, and are mostly found incidentally during routine colonoscopies or other explorations [2]. Clinical signs and symptoms depend on the size and location of the lesion. These tumors may range in dimensions from 2 mm to 30 cm [1], generally presenting symptoms once the size exceeds 2 cm [2]. Patients may present with a range of unspecific symptoms that may resemble malignant pathologies and symptoms such as abdominal pain, diarrhea, constipation, hematochezia, and anal prolapse, and in rare instances, it may lead to complications such as bowel obstruction or intussusception.

A crucial discussion point with regard to optimal therapeutic management is the discrimination of the cause of intussusception, whether it is benign, malignant, or idiopathic [4]. Such diagnostic differentiation is not achieved with imaging tests and is only reliable via histological analysis, leading to the discussion of which is the most appropriate therapeutic procedure in a rare case of a colonic lipoma causing bowel intussusception. Therapeutic options range from local excision (enucleation) to segmental colonic resection, being performed either intraluminally or laparoscopically [5]. Low rectal lipomas can also be removed through the anus [2].

The most conservative option is the simple removal of the tumor or enucleation, performed both endoscopically or surgically [1]. These techniques allow for an improvement in pain control, shorter hospitalization, and faster recovery, but bleeding and perforation risk are higher in lipomas >2 cm or with a wide base [5-7]. Therefore, considering how the lipoma in our case exceeded 2 cm, it has an increased risk of bleeding or perforation, excluding this therapeutic option. This decision is further supported by Alvarez-Bautista et al. [4] and Jiang et al. [8], explaining that endoscopic resection should not be performed in tumors >2 cm; however, surgical resection is appropriate when the lipoma is bigger than 4 cm or has an associated intussusception with symptoms. The decision to exclude the therapeutic option of local excision of the tumor was based on the uncertainty of the diagnosis complicated by the intussusception [9].

In our particular case, we performed an elective oncologic sigmoid resection due to inherent uncertain diagnosis, the size of the lesion, the potential risks of endoscopic treatment, and some cases reported with pseudosarcomatous changes in suspected lipomas [10-12].

## Conclusions

As the presentation of colonic lipoma with associated intussusception causing transanal protrusion and colonic obstruction is uncommon, a complete preoperative study including blood analysis and imaging techniques should be performed and must be considered in the differential diagnosis of bowel tumors. Taking into consideration that the lipoma measured more than 2 cm, the tumor's location, the associated symptoms, and the additional complication of the intussusception leading to an unclear preoperative diagnosis, a surgical resection was considered the treatment of choice for this particular case.
